# Causal relationship between gut microbiota and pathological scars: a two-sample Mendelian randomization study

**DOI:** 10.3389/fmed.2024.1405097

**Published:** 2024-07-02

**Authors:** Huidi Shucheng, Jiaqi Li, Yu-ling Liu, Xinghan Chen, Xian Jiang

**Affiliations:** ^1^Department of Dermatology, West China Hospital, Sichuan University, Chengdu, China; ^2^Laboratory of Dermatology, Clinical Institute of Inflammation and Immunology, Frontiers Science Center for Disease-related Molecular Network, West China Hospital, Sichuan University, Chengdu, China; ^3^Med-X Center for Informatics, Sichuan University, Chengdu, China; ^4^Research Institute of Tissue Engineering and Stem Cells, Nanchong, China; ^5^Department of Burns and Plastic Surgery, West China Hospital, Sichuan University, Chengdu, China

**Keywords:** Mendelian randomization, gut microbiota, keloids, hypertrophic scars, pathological scars

## Abstract

**Background:**

Pathological scars, including keloids and hypertrophic scars, represent a significant dermatological challenge, and emerging evidence suggests a potential role for the gut microbiota in this process.

**Methods:**

Utilizing a two-sample Mendelian randomization (MR) methodology, this study meticulously analyzed data from genome-wide association studies (GWASs) relevant to the gut microbiota, keloids, and hypertrophic scars. The integrity and reliability of the results were rigorously evaluated through sensitivity, heterogeneity, pleiotropy, and directionality analyses.

**Results:**

By employing inverse variance weighted (IVW) method, our findings revealed a causal influence of five bacterial taxa on keloid formation: class *Melainabacteria*, class *Negativicutes*, order *Selenomonadales*, family *XIII*, and genus *Coprococcus2*. Seven gut microbiota have been identified as having causal relationships with hypertrophic scars: class *Alphaproteobacteria*, family *Clostridiaceae1*, family *Desulfovibrionaceae*, genus *Eubacterium coprostanoligenes group*, genus *Eubacterium fissicatena group*, genus *Erysipelotrichaceae UCG003* and genus *Subdoligranulum*. Additional sensitivity analyses further validated the robustness of the associations above.

**Conclusion:**

Overall, our MR analysis supports the hypothesis that gut microbiota is causally linked to pathological scar formation, providing pivotal insights for future mechanistic and clinical research in this domain.

## Introduction

Wound healing is a multifaceted and dynamic process, governed by a balance of numerous regulatory pathways. A deviation from this balance can lead to pathological scars, chiefly hypertrophic scars and keloids, characterized by excessive extracellular matrix deposition and abnormal fibroblast behavior (1, 2). The profound impact of pathological scars extends beyond their physical appearance, encompassing cosmetic concerns, functional impairments such as contractures, and subjective symptoms including pruritus and pain (3). Collectively, these aspects highlight the far-reaching effects of pathological scars, with significantly influencing various facets of patient well-being (3).

The intricate mechanisms underlying the formation of these scars involve both local and systemic factors, including genetic predispositions (4). Recent advancements in dermatological research have highlighted the potential influence of the gut microbiota on skin health, particularly in the context of inflammatory disorders (5). This has led to the exploration of the “gut-skin axis,” a concept describing the complex interactions between the gut microbiota and skin health (5). Alterations in the gut microbiota have been linked to skin inflammation and the manifestation of various skin diseases, including rosacea, psoriasis, and atopic dermatitis (6–8). The gut-skin axis involves complex interactions where the gut microbiome impacts skin health through immune modulation, metabolic functions, and hormonal pathways. Gut bacteria influence immune responses, producing anti-inflammatory mediators that reduce skin inflammation (9). They also metabolize dietary components into bioactive metabolites like short-chain fatty acids, enhancing skin barrier function and hydration (10). Additionally, the gut microbiome affects nutrient absorption, essential for skin health (5). Hormonal pathways are influenced as gut bacteria modulate hormones such as cortisol and serotonin, impacting conditions like acne and psoriasis (11). Therapeutic approaches include probiotics and dietary modifications to restore gut microbiome balance, offering potential for improved skin health (12). Notably, a recent study revealed distinct differences in the gut microbiota composition between individuals with normal scars and those with pathological scars (13). This emerging evidence suggests that the gut microbiota may also play a role in the development of pathological scars, adding a new dimension to our understanding of skin disorders. A pivotal question that arises from these observations is whether the relationship between gut microbiota imbalance and the onset or exacerbation of pathological scars is merely observational or indicative of a direct causal influence. Addressing this question is challenging due to the multifaceted nature of gut microbiota, which is influenced by a range of factors such as age, sex, body mass index, and even environmental factors. These variables not only affect the composition of the gut microbiota but also complicate the task of assembling a diverse and representative sample for research. Furthermore, ethical considerations and practical limitations pose significant hurdles in conducting clinical trials to investigate the causal links between the gut microbiota alterations and pathological scar development.

To address these challenges, our study employs a two-sample Mendelian randomization (MR) approach. MR utilizes genetic variants as instrumental variables to establish causal relationships between exposures (such as variations in the gut microbiota) and outcomes (such as pathological scars) (14). This method offers a strategic advantage over traditional randomized controlled trials (RCTs) and observational studies, as it reduces the confounding biases and ethical concerns associated with direct manipulation of the gut microbiota in human subjects (15). By leveraging this approach, our study aims to dissect the potential causal relationship between gut microbiota and the development of pathological scars. The findings from this research could significantly enhance our understanding of the gut-skin axis and its implications for skin health, potentially leading to novel therapeutic strategies for managing pathological scars.

## Methods

### Study design

**Figure 1:**
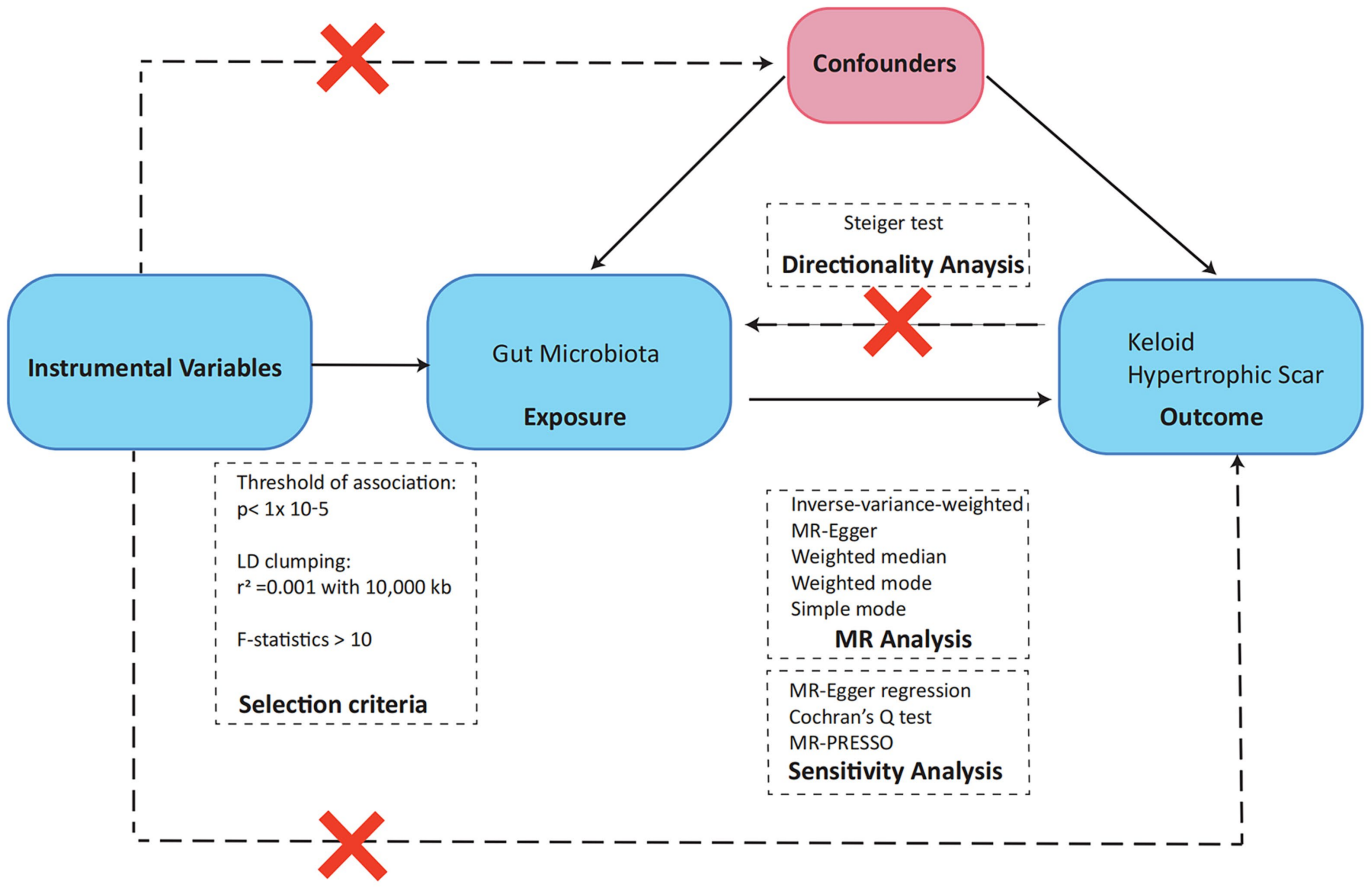
Overview of the Mendelian randomization analysis and three main assumptions.

Our study’s analytical framework, depicted in Figure 1, employs a two-sample MR design to investigate the potential causal relationships between gut microbiota and pathological scar formation. This approach is underpinned by three fundamental hypotheses, each aligning with core MR principles and collectively crucial in elucidating the connection between the gut microbiota and pathological scars. The initial hypothesis centers on the representativeness of the genetic instruments utilized in our MR analysis. These instruments must accurately reflect the exposure of interest, which in this case is the gut microbiota. This is essential for reliably measuring the impact of gut microbiota variations on the development of pathological scars. Our second hypothesis pertains to the independence of these genetic instruments from confounding factors. This aspect is critical for mitigating the risk of spurious associations, thereby bolstering the validity of our causal inferences. By ensuring this independence, we strengthened the credibility of our findings in linking gut microbiota alterations to pathological scars. Finally, the third hypothesis, known as the exclusion restriction hypothesis, plays a pivotal role in establishing a direct causal pathway between changes in gut microbiota and the manifestation of pathological scars. This hypothesis is integral to reinforcing the causal interpretation derived from our MR analysis, as it asserts that the observed associations are not influenced by external factors unrelated to the gut microbiota-pathological scar nexus (16).

### Data source

In this study, genetic variants for the gut microbiota were derived from an extensive genome-wide association study (GWAS) meta-analysis (17). This analysis included a predominantly European cohort of 18,340 participants across 24 separate cohorts. Post-imputation, the analysis covered 5,717,754 SNPs. In terms of the gut microbiota, the original study categorized it into 257 taxa across six taxonomic levels: phylum [p], class [c], order [o], family [f], and genus [g]. For our microbial quantitative trait locus (mbQTL) mapping analysis, this number was reduced due to quality control and filtering, excluding taxa with low prevalence or abundance. Out of the remaining 211 taxa, we excluded 15 unknown taxa and ultimately included 196 taxa. This selection comprised 9 phyla, 16 classes, 20 orders, 32 families, and 119 genera.

Additionally, GWAS summary statistics for hypertrophic scars were obtained from the latest R10 release data of the FinnGen consortium. This dataset comprises 1,641 documented hypertrophic scar cases, juxtaposed with a control cohort of 385,559 individuals. The GWAS summary statistics for keloids were extracted from a meta-analysis conducted by Sakaue et al. (18). The present study included 668 documented keloid cases with 481,244 control cases and identified a total of 24,197,210 SNPs to explore their potential associations sdwith keloids. The utilization of these diverse and comprehensive sources of data will undoubtedly facilitate a meticulous and thorough investigation into delineating the potential causal relationships that exist between the gut microbiota and pathological scars. No obvious overlap between exposures and outcomes.

### Instrumental variable selection

To identify potential instrumental variables (IVs) for our study, we first concentrated on single nucleotide polymorphisms (SNPs) associated with gut bacterial taxa, adhering to the genome-wide significance benchmark of *p* < 1.0 × 10–5. The selection of appropriate IVs hinged on meeting stringent quality control standards, which included establishing a linkage disequilibrium (LD) threshold for clumping at *r*^2^ < 0.001 within a 10,000 kb window, essential for minimizing LD impact and ensuring SNP independence. Additionally, we aligned the effect estimates of exposure and outcome variants, discarded SNPs with incompatible alleles or palindromic characteristics, and selected only SNPs present for all evaluated traits as IVs, avoiding the use of proxies for absent traits. Furthermore, we conducted comprehensive examinations of each SNP via PhenoScanner V2^1^ to determine whether the SNPs influenced outcomes exclusively through their exposure. Finally, we evaluated the robustness of our chosen instruments by calculating the F statistic using the formula: *F* = (β/SE)^2^, where β represents the effect size and SE the standard error, adhering to an *F* > 10 criterion to mitigate bias toward weak IVs (19).

### MR analysis

Our foundational analysis incorporated a comprehensive array of MR techniques to establish causal effects. Central to this was the inverse-variance weighted (IVW) method, which formed the core of our analysis, and was augmented by additional methods, including the simple model, weighted model, weighted median, and MR-Egger methods. These factors collectively enabled a thorough evaluation of causal relationships. To address the potential bias due to pleiotropy, we focused on the intercept term of MR-Egger regression. A near-zero intercept term suggests the absence of horizontal pleiotropy in our bidirectional MR approach, a crucial aspect in determining the validity of the SNP under investigation (20). To further investigate horizontal pleiotropy, where a single genetic variant may affect multiple traits and complicate causal inference, we employed the MR-PRESSO global test (21). We also meticulously assessed heterogeneity within the IVW method using Cochran’s Q statistics and funnel plots, tools that illuminate the consistency and reliability of our results. Given the extensive scope of hypothesis testing, the Benjamini-Hochberg (BH) procedure was rigorously applied to adjust for multiple comparisons in our analysis. The results with a false discovery rate *p* value (PFDR) below 0.1 were considered significant, setting a high bar was used to indicate statistical significance. However, outcomes with a *p* value less than 0.05 but a PFDR above 0.1, while not meeting this stringent criterion, were still noted for their nominal significance, indicating potential emerging trends meriting further investigation. Finally, the MR Steiger test was employed to rigorously determine the direction of causality between the exposure and the outcome, offering essential insight into the causative pathways involved in our study. All analyses were performed using the package “Two-Sample-MR” (version 0.5.6), “MR-PRESSO” (version 1.0) in R (version 4.3.0).

## Results

**Figure 2:**
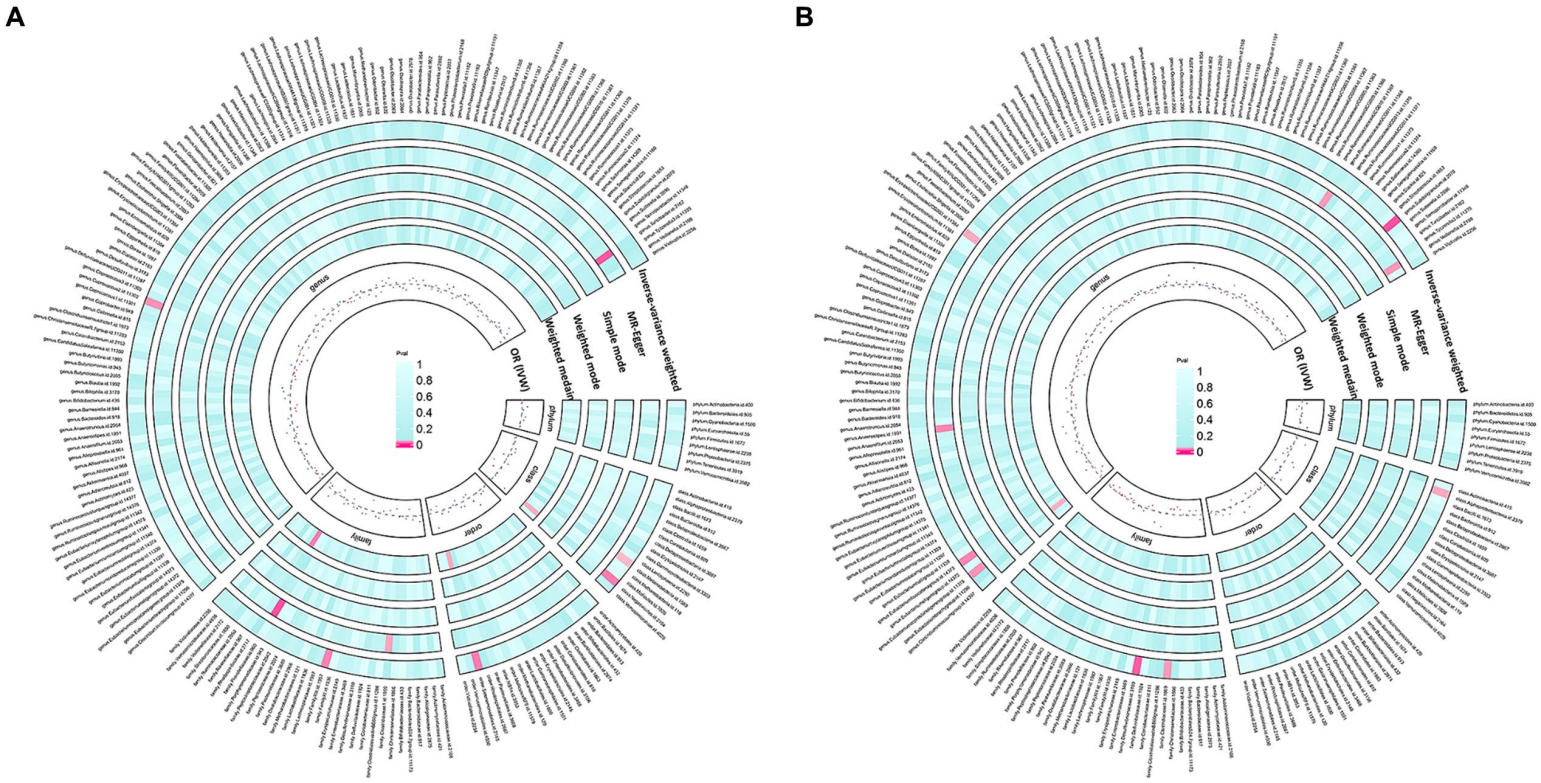
Preliminary MR estimates of gut microbiota’s association with keloids **(A)** and hypertrophic scars **(B)**.

**Figure 3:**
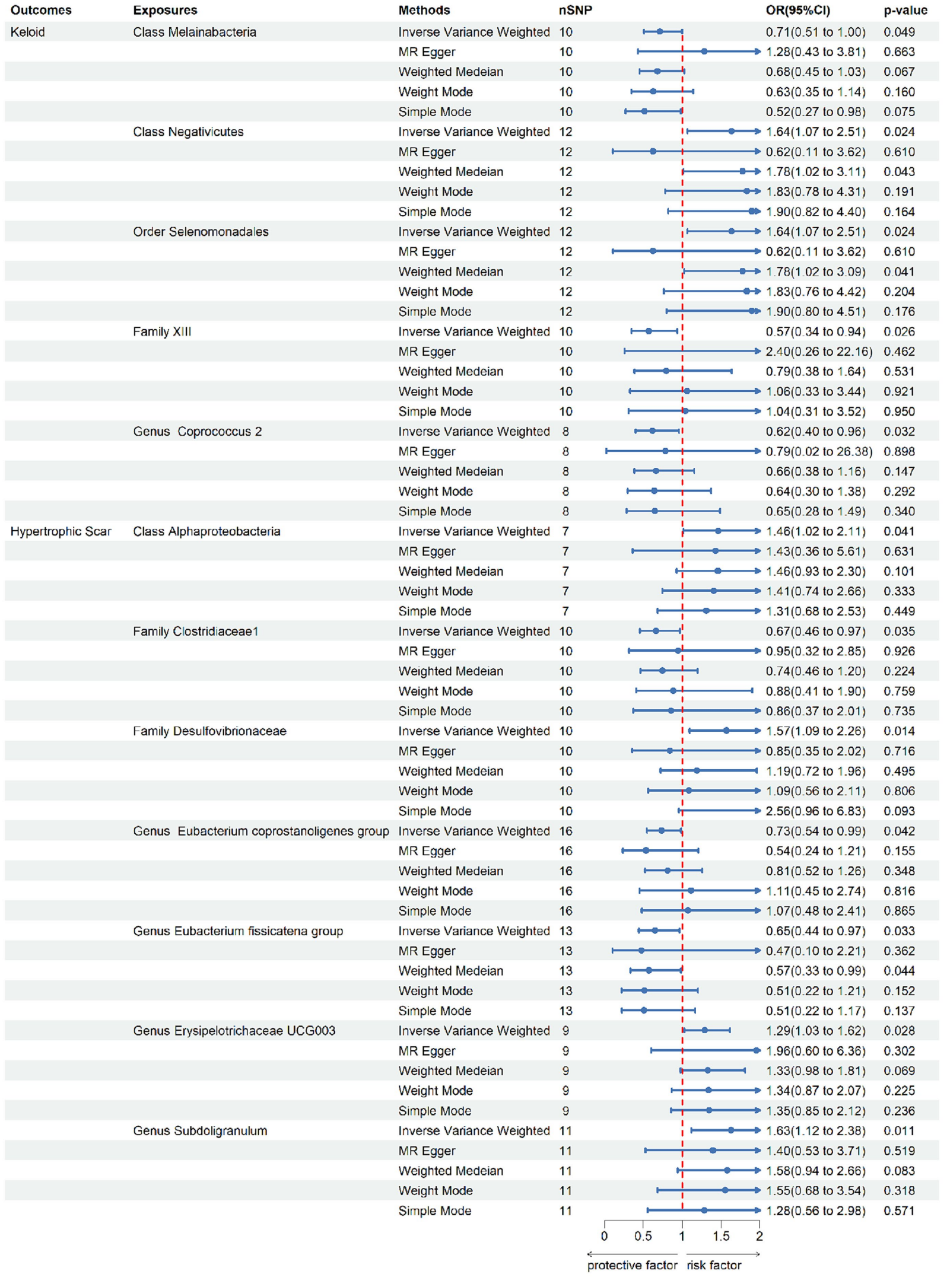
Forest plot of the causal association between gut microbiota and pathological scars.

According to the IV selection, a total of 2078 SNPs were used as IVs for SNPs associated with the gut microbiota. We explored the association between the gut microbiota and pathological scars using five MR methods (Figure 2; Supplementary Tables S1, S2). All the F-statistics of the IVs were greater than 10, which indicated that weak instrument bias was unlikely. The IVW methods highlighted five bacterial genera: class *Melainabacteria* (OR = 0.71, 95% CI: 0.51–1.00, *p* = 0.046), class *Negativicutes* (OR = 1.64, 95% CI: 1.07–2.51, *p* = 0.024), order *Selenomonadales* (OR = 1.64, 95% CI: 1.07–2.51, *p* = 0.024), family *XIII* (OR = 0.57, 95% CI: 0.35–0.94, *p* = 0.026) and genus *Coprococcus2* (OR = 0.62, 95% CI: 0.40–0.96, *p* = 0.032) (Figure 3). These factors play a protective role by preventing the onset of keloids. Additionally, our examination revealed seven gut microbiota taxa exerting significant causal effects on hypertrophic scars, and a range of taxa from broad phyla to specific genera was identified: class *Alphaproteobacteria* (OR = 1.46, 95% CI: 1.02–2.11, *p* = 0.041), family *Clostridiaceae1* (OR = 0.67, 95% CI: 0.46–0.97, *p* = 0.035), family *Desulfovibrionaceae* (OR = 1.57, 95% CI: 1.09–2.26, *p* = 0.014), genus *Eubacterium coprostanoligenes group* (OR = 0.73, 95% CI: 0.55–0.99, *p* = 0.042), genus *Eubacterium fissicatena group* (OR = 0.65, 95% CI: 0.44–0.97, *p* = 0.033), genus *Erysipelotrichaceae UCG003* (OR = 1.29, 95% CI: 1.03–1.62, *p* = 0.028) and genus *Subdoligranulum* (OR = 1.64, 95%CI: 1.07–2.51, *p* = 0.024) (Figure 3). The above results are depicted by scatter plots in Supplementary Figure S1 and illustrated through forest plots for causal effects of gut microbiota on keloids and hypertrophic scars with individual SNPs in Supplementary Figure S2. Despite the observed associations, no MR outcomes met the FDR correction threshold for multiple testing (Q_FDR_ < 0.1). Nevertheless, with *p*-values < 0.05, these results hold nominally significant (Supplementary Table S1).

In our rigorous analysis of data homogeneity, we utilized Cochrane’s Q test and observed no significant heterogeneity among the selected single nucleotide polymorphisms (SNPs), with p-values exceeding 0.05, as detailed in Table 1. This lack of heterogeneity underscores the consistency of the SNP selection for our study. Additionally, we conducted comprehensive pleiotropy assessments using both the MR Egger test and the MR-PRESSO analysis. Pleiotropy occurs when a genetic variant influences multiple traits, which can bias the results of a Mendelian Randomization study. The MR Egger intercept provides evidence of directional pleiotropy if the intercept significantly deviates from zero. MR-PRESSO identifies and corrects for outliers that may bias the MR estimates. These tests consistently indicated the absence of pleiotropy, with all *p* values again exceeding the 0.05 threshold (Table 2). Crucially, our study employed MR Steiger directionality tests, which uniformly indicated a strong causal relationship running from the gut microbiota to pathological scars across all evaluated outcomes. This finding, detailed in Supplementary Table S3, solidifies the direction of the causal pathway in our research. This consistent demonstration of a causal direction not only reinforces the robustness of our findings but also highlights the potential impact of the gut microbiota on the formation of pathological scars.

**Table 1:** Results of heterogeneity analysis using Cochran’s Q test for causal associations between gut microbiota and pathological scars.

Exposure	Outcome	IVW		MR-Egger	
		*Q*	*p* value	*Q*	*p* value
Class *Melainabacteria*	Keloids	12.34	0.19	10.68	0.22
Class *Negativicutes*		3.73	0.98	2.51	0.99
Order *Selenomonadales*		3.73	0.98	2.51	0.99
Family *XIII*		9.10	0.42	7.41	0.49
Genus *Coprococcus2*		2.76	0.91	2.74	0.84
Class *Alphaproteobacteria*	Hypertrophic scars	1.48	0.96	1.48	0.92
Family *Clostridiaceae1*		4.40	0.88	3.95	0.86
Family *Desulfovibrionaceae*		7.90	0.54	5.55	0.70
Genus *Eubacterium coprostanoligenes group*		7.80	0.80	7.62	0.75
Genus *Eubacterium fissicatena group*		5.76	0.67	5.27	0.63
Genus *Erysipelotrichaceae UCG003*		12.28	0.66	11.61	0.64
Genus *Subdoligranulum*		6.88	0.74	6.76	0.66

IVW, inverse-variance weighted.

**Table 2:** Pleiotropy assessment using Egger intercept analysis and MR-PRESSO.

Exposure	Outcome	MR egger-intercept		*p* value of MR- PRESSO global
		Intercept	*p* value	
Class *Melainabacteria*	Keloids	−0.065	0.297	0.238
Class *Negativicutes*		0.061	0.294	0.984
Order *Selenomonadales*		0.061	0.294	0.975
Family *Family XIII*		−0.096	0.229	0.426
Genus *Coprococcus2*		−0.017	0.898	0.904
Class *Alphaproteobacteria*	Hypertrophic Scars	0.002	0.972	0.958
Family *Clostridiaceae1*		−0.028	0.522	0.892
Family *Desulfovibrionaceae*		0.053	0.163	0.552
Genus *Eubacterium coprostanoligenes group*		0.020	0.681	0.816
Genus *Eubacterium fissicatena group*		−0.055	0.503	0.681
Genus *Erysipelotrichaceae UCG003*		0.030	0.428	0.636
Genus *Subdoligranulum*		0.012	0.743	0.769

MR Presso Global, Mendelian Randomization Pleiotropy RESidual Sum and Outlier Global Test.

## Discussion

The complex interplay between the gut microbiota and various health outcomes has been a focal point of numerous investigations in recent years. Our study focused on this ever-evolving frontier to delineate the causal relationships between the gut microbiota and pathological scars. We identified five and seven distinct gut microbiota taxa causally linked to keloids and hypertrophic scar formations, respectively. Our findings underscore a promising direction for understanding the etiopathogenesis of pathological scars and open new vistas for potential therapeutic interventions.

The complex interplay between the gastrointestinal tract and skin, known as the gut-skin axis, is pivotal for sustaining skin homeostasis (22). Central to this interaction is the role of the gut microbiome in modulating both systemic and local inflammation through immune system engagement. In the gut, microbial communities maintain barrier integrity by metabolizing complex polysaccharides into vital vitamins and short-chain fatty acids (SCFAs), such as butyrate and propionate (23, 24). These substances are integral to reinforcing intestinal barrier strength and minimizing permeability (24). Furthermore, the presentation of commensal antigens by dendritic cells (DCs) facilitates the differentiation of gut commensal bacteria-specific Tregs, IgA-producing B cells, and Th17 cells (22). This process is vital for maintaining the equilibrium of the gut microbiota and preventing bacterial entry into the bloodstream, thereby contributing to skin homeostasis. Skin inflammation can be influenced by minor alterations in specific bacterial species of the intestinal microbiome, potentially leading to disorders such as acne, alopecia areata, atopic dermatitis and psoriasis (5). For instance, *Faecalibacterium prausnitzii* helps protect against psoriasis by competitively inhibiting the colonization of pathogenic skin flora and producing SCFAs, which modulate inflammatory responses (25), and *Lactobacillus casei* helps reduce skin inflammation by decreasing the number of cytotoxic CD8+ T cells, thereby modulating the immune response and promoting skin health (26). In this study, it was discovered that the microbiota identified as potentially protective against pathological scars may exert its protective effects on pathological scars through similar pathways. The *Coprococcus 2* genus, classified as a gram-positive bacteria, is instrumental in producing butyrate, a compound with anti-inflammatory properties that notably inhibits nuclear factor κB, mitigates reactive oxygen species, and fosters both cell differentiation and intestinal health (25). A decrease in *Coprococcus 2* levels, leading to a consequential decrease in butyrate within the gastrointestinal tract, may adversely affect immune functionality, potentially precipitating the development of keloids. *Erysipelotrichaceae UCG-003*, another butyrate-producing bacterium, is essential for colonic epithelial integrity, and its abundance is notably lower in patients with neurological disorders and lung cancer than in healthy individuals (27–29). This study also revealed that other gut microbiota taxa can influence the formation of pathological scars as protective factors or risk factors, but their specific mechanisms require further investigation.

Emerging evidence increasingly underscores a significant correlation between pathological scars and the microbiota. Regarding the skin microbiota, studies have identified dysbiosis predominantly characterized by *S. aureus*, which is linked to chronic inflammation and subsequent hypertrophic scar development (30). The topical application of probiotics may also represent a promising approach for the prevention of pathological scars. Lombardi et al.’s research revealed that *S. thermophilus* lysate markedly reduces the primary mediators and activities involved in the abnormal activation of myofibroblasts triggered by TGF-β1 under fibrotic conditions. This treatment notably decreased the expression of α-SMA, fibronectin, and collagen-I, while also diminishing the collagen contraction ability of activated dermal fibroblasts (31). Focusing on the gut microbiota, Li et al. utilized 16S rRNA gene sequencing to investigate generalizable microbial signatures associated with pathological scars and their relationship with the gut microbiota. Their findings highlighted distinctions in alpha and beta diversity at the phylum and genus levels between patients with physiological and pathological scars, indicating a dysregulated gut microbiota in individuals with pathological scars (13). Notably, a significantly greater presence of *Subdoligranulum* was observed in the gut microbiota of patients with pathological scars than in that of patients with physiological scars. Our research further corroborates this finding, establishing a causal link between increased *Subdoligranulum* abundance and the development of hypertrophic scars, thus offering a mechanistic insight into previous observations. However, our findings present some divergences from those reported in Li′s study. This disparity may arise from several factors. First, Li et al. did not distinguish keloids from hypertrophic scars within pathological scars, and our study results indicate that the impact of the gut microbiota on keloids and hypertrophic scars is not identical. Second, our research sample comprised individuals of European descent, in contrast to the East Asian population in Li′s study. Third, the methodological strengths of our MR study include its capacity to explore causal relationships between exposure and outcomes while effectively controlling for confounders, which pose significant challenges in observational settings. Additionally, we have noted recent studies exploring the causal relationship between hypertrophic scars and gut microbiota by MR (32). Our research uniquely investigates the causal relationships between gut microbiota and both hypertrophic scars and keloids. Interestingly, we found distinct causal relationships for these two types of pathological scars with gut microbiota, offering new perspectives on their pathogenesis.

Our MR analysis revealed a subset of the gut microbiota with protective effects against pathological scars, most of which are directly or indirectly related to the production of SCFAs. For instance, the genus *Coprococcus*, a classic butyrate-producing microbial group, has been shown to have protective effects against a variety of diseases, including atopic disease and Parkinson’s disease (33, 34). Within the genus *Eubacterium*, groups such as *Eubacterium coprostanoligenes* and *Eubacterium fissicatena* are involved in cholesterol metabolism and are capable of producing SCFAs during the degradation of cholesterol (35). Additionally, certain microbial groups within family XIII and family *Clostridiaceae 1*, such as *Clostridium*, are well-known producers of butyric acid and other SCFAs (36, 37). The relationships among the gut microbiota, SCFAs, and the formation of pathological scars, such as keloids and hypertrophic scars, have emerged as a focal point of interest in understanding the complex mechanisms of fibrosis. The lipid hypothesis of pathological scar pathogenesis has emphasized the role of triglycerides, cholesterol, and unsaturated fatty acids, while investigations into SCFAs, such as butyric acid, isobutyric acid, malonic acid, isovaleric acid, and valeric acid, have been comparatively limited. Recent studies underscore the significance of these SCFAs, which are produced by the anaerobic fermentation of dietary fibers by colonic bacteria, and play crucial roles in various cellular processes, including apoptosis, proliferation, and differentiation, largely through their histone deacetylase inhibitor activity (38). SCFAs are present at lower concentrations in keloid tissues than in normal skin, suggesting disrupted metabolic equilibrium (39, 40). This reduction, particularly of butyric acid, suggests a compromised antifibrotic defense mechanism, potentially exacerbating scar pathogenesis through the deregulation of keloid-derived fibroblast activity. Such deregulation could culminate in unchecked cellular proliferation and excessive collagen deposition. Indeed, *in vitro* experiments have shown that butyrate can attenuate fibroblast proliferation and modulate inflammatory responses, in a concentration-dependent manner: lower concentrations of butyrate promote cellular proliferation, while higher concentrations of butyrate promote apoptosis (41, 42). Pathological scars are also intricately associated with inflammation and the connection between SCFAs and inflammation is highlighted by their regulatory effect on mast cells and the production of inflammatory mediators. Specifically, butyrate and propionate have been shown to inhibit the activation of mast cells, potentially mitigating inflammation-driven scar formation (43). In summary, the relationships among the gut microbiota, SCFAs, and pathological scars present a complex yet logically coherent narrative that encompasses metabolic, immunological, and cellular perspectives. Our investigation, through a bioinformatics approach, contributes novel insights into this intricate relationship, enriching the existing body of knowledge with additional reference points for further understanding the underlying mechanisms involved.

This study, while insightful, is subject to several limitations. First, it’s important to acknowledge that the results did not adhere to the stringent FDR correction threshold. Despite not meeting this rigorous standard, the nominal significance of the findings warrants consideration. Second, our analysis did not account for sex differences, a factor that could have influenced the outcomes, particularly given known sex-specific variations in the skin microbiome of burn scars (44). Third, the genetic studies we referenced were limited to populations of European ancestry, potentially introducing stratification bias. This is especially relevant considering the higher incidence of pathological scars among African and Asian individuals, which may limit the applicability of our results to these groups. Fourthly, our study has only validated the causal relationship between gut microbiota and pathological scars at a bioinformatics level; these relationships still lack validation through molecular biology and clinical trials. Finally, our analysis was limited by the taxonomic resolution of the exposure dataset, which was limited to the genus level, thereby restricting our ability to investigate species-level relationships.

## Conclusion

In this study, we conducted a comprehensive two-sample MR analysis utilizing publicly accessible GWAS summary-level data, to evaluate the causal influence of the gut microbiota on the development of pathological scars. Our MR analysis robustly supports the hypothesis that the gut microbiota is causally linked to pathological scar formation, providing pivotal insights for future mechanistic and clinical research in this domain.

## Data availability statement

The original contributions presented in the study are included in the article/Supplementary material, further inquiries can be directed to the corresponding author.

## Ethics statement

In accordance with local legislation and institutional requirements, ethical review and approval for the study involving human participants was not necessary. This article does not include any potentially identifiable images or data.

## Author contributions

HS: Validation, Methodology, Conceptualization, Writing – review & editing, Writing – original draft. JL: Writing – original draft, Writing – review & editing, Conceptualization, Data curation, Visualization. Y-lL: Visualization, Writing – review & editing, Writing – original draft. XC: Validation, Methodology, Data curation, Conceptualization, Writing – review & editing, Writing – original draft. XJ: Conceptualization, Writing – review & editing, Writing – original draft.

## Footnotes

^1^ http://www.phenoscanner.medschl.cam.ac.uk/
