# Supplementary material for: Causal relationship between gut microbiota and pathological scars: a two-sample Mendelian randomization study
Source: Front Med (Lausanne). 2024 Jul 2;11:1405097. doi: 10.3389/fmed.2024.1405097 (PMC11250559; doi:10.3389/fmed.2024.1405097)
Supplement: Supplementary file 4 [file Data_Sheet_1.PDF]

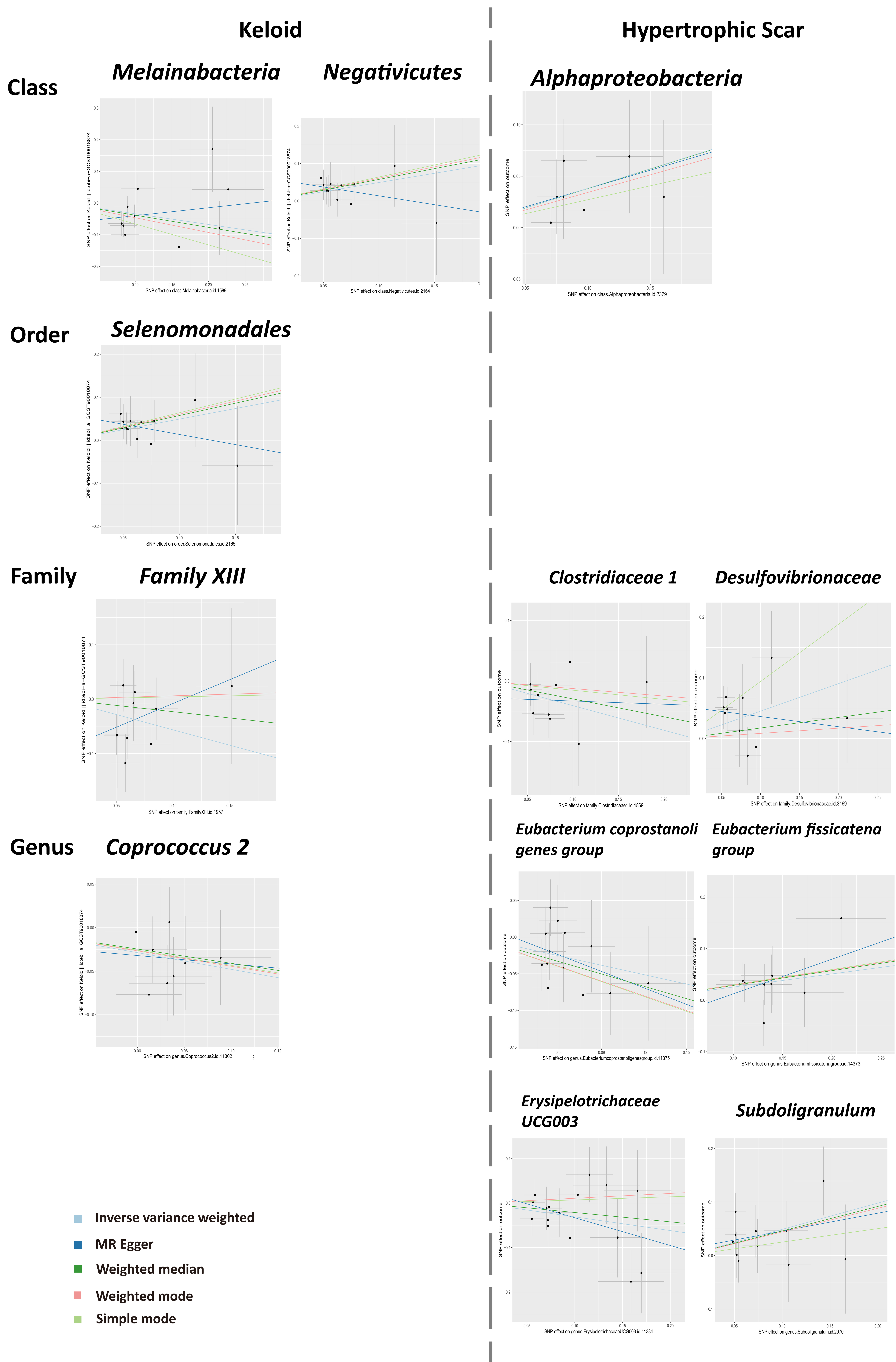
Keloid
Hypertrophic Scar
Class
Melainabacteria
Negativicutes
Alphaproteobacteria
SNP effect on Keloid || id:ebi-a-GCST90018874
SNP effect on class.Melainabacteria.id.1589
SNP effect on class.Negativicutes.id.2164
SNP effect on outcome
SNP effect on class.Alphaproteobacteria.id.2379
Order
Selenomonadales
SNP effect on order.Selenomonadales.id.2165
Family
Family XIII
SNP effect on family.FamilyXIII.id.1957
Clostridiaceae 1
SNP effect on family.Clostridiaceae1.id.1869
Desulfovibrionaceae
SNP effect on family.Desulfovibrionaceae.id.3169
Genus
Coprococcus 2
SNP effect on genus.Coprococcus2.id.11302
Eubacterium coprostanoli genes group
SNP effect on genus.Eubacteriumcoprostanoligenesgroup.id.11375
Eubacterium fissicatena group
SNP effect on genus.Eubacteriumfissicatenagroup.id.14373
Erysipelotrichaceae UCG003
SNP effect on genus.ErysipelotrichaceaeUCG003.id.11384
Subdoligranulum
SNP effect on genus.Subdoligranulum.id.2070
Inverse variance weighted
MR Egger
Weighted median
Weighted mode
Simple mode
