# Supplementary material for: Causal relationship between gut microbiota and pathological scars: a two-sample Mendelian randomization study
Source: Front Med (Lausanne). 2024 Jul 2;11:1405097. doi: 10.3389/fmed.2024.1405097 (PMC11250559; doi:10.3389/fmed.2024.1405097)
Supplement: Supplementary file 5 [file Data_Sheet_2.PDF]

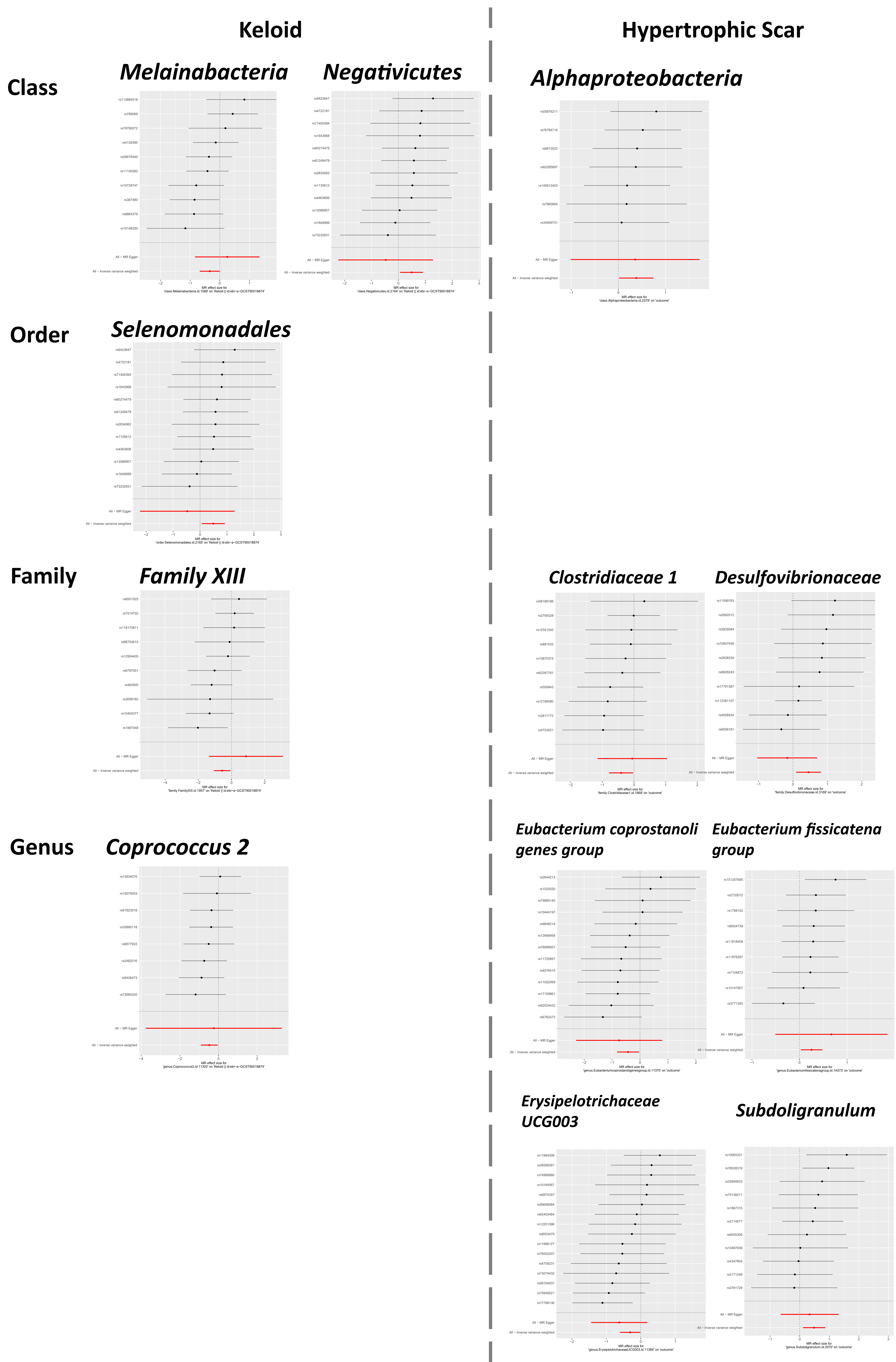
Keloid
Hypertrophic Scar
Class
Melainabacteria
Negativicutes
Alphaproteobacteria
Order
Selenomonadales
Family
Family XIII
Clostridiaceae 1
Desulfovibrionaceae
Genus
Coprococcus 2
Eubacterium coprostanoli genes group
Eubacterium fissicatena group
Erysipelotrichaceae UCG003
Subdoligranulum
